# Improvement of Adherence to the Mediterranean Diet through a Nutrition Education Teaching Pack for Teachers within the “School Fruit Scheme” Program: An Italian Long-Term Trial in School Children

**DOI:** 10.3390/nu16132057

**Published:** 2024-06-27

**Authors:** Romana Roccaldo, Laura Censi, Laura D’Addezio, Sibilla Berni Canani, Laura Gennaro

**Affiliations:** Council for Agricultural Research and Economics, Research Centre for Food and Nutrition, 00178 Rome, Italylaura.daddezio@crea.gov.it (L.D.);

**Keywords:** KIDMED, school children, nutrition education, effectiveness, Italy

## Abstract

A previous short time span study related to the effectiveness of a teaching pack (TP) in improving the adherence to the Mediterranean Diet (MD) showed positive results. The present study was aimed at investigating and confirming those results, with a follow up data collection, in the same sample, a year after the baseline intervention. Pre- and post-intervention assessments were conducted. Weight and height were measured. Eating patterns/lifestyle were assessed by the KIDMED test and questionnaires. Thirteen schools in three areas with low, medium and high prevalence of overweight/obesity (North, Center and South respectively) were involved, with a representative baseline cluster sample of 494 fourth class children (8–10 years old) in 2015. An intervention group and a control group were recruited in each school; the intervention group (n = 395) got the intervention, the control group (n = 99) did not. The children’s KIDMED score changes were the main outcome measures. Differences in percentages of adherence and in yes/no answers on the KIDMED test, at baseline and after one year, for both the intervention and the control groups, were assessed through contingency tables and statistical tests. Improvements in the high and low adherence rates to MD were observed (high adherence: 24.4% to 43.3%; low adherence: 15.0% to 3.9%, *p* < 0.0001). The percentages of subjects with optimal adherence improved in both sexes (females: 25.5% to 49.5%, *p* < 0.0001; males: 23.1% to 36.6%, *p* < 0.0001) in all the geographical areas and ponderal status classes. Accompanying free distribution of fruit and vegetables with a nutritional intervention led by trained teachers with a cross-curricular approach can be successful in promoting healthy eating in children.

## 1. Introduction

Proper and healthy diet with an adequate lifestyle is fundamental for children to grow up in a healthy way with a global feeling of well-being, satisfying scholastic performance, physical health throughout life [1,2,3] and for sustaining their immune systems [4].

Conversely, an unhealthy diet, with small fruit and vegetable (FV) intakes and high in sugar, saturated fats and salt together with little/lack of exercise, is among the main risk factors for chronic diseases, which have increased continuously worldwide in the recent past and represent the majority of the global deaths [5,6]. The exposure to the above risk factors frequently begins in the early phase of life, with serious consequences in later years into adulthood [7,8,9,10]. Therefore, it is fundamental to promote healthy patterns early in life. The school environment is reckoned to be the ideal context to educate children about healthy eating habits and lifestyle and to limit exposure to unhealthy foods and drinks [6,11,12,13], and the best way to involve copious numbers of children, school personnel, family and community members [14,15]. Schools are where children spend a wide proportion of their days, and school years (5 to 15 years of age) see all the changes due to growth and development. Good nutrition is fundamental to support all those changes and to overcome early deficits [16].

Most interventions carried out at school are aimed to increase children’s intake of FVs. In Italy, as in many other countries, the national dietary recommendations of FVs [17,18,19,20] are not being followed, despite the numerous health benefits associated with FV consumption [21,22,23].

FVs are also fundamental components of the Mediterranean Diet (MD) (together with pulses, cereals, nuts and olive oil), an adequate and very healthy dietary pattern well known for its numerous benefits [24,25,26,27], which can be evident even in childhood [28,29,30].

Unfortunately, in spite of all the benefits, a tendency to abandon the Mediterranean lifestyle has been observed in Italy as well as in other Mediterranean countries, especially among young people, who prefer foods rich in sugar, saturated fats, and salt, and poor in FV [24,31,32,33,34,35].

In the scholastic year 2009–2010 the School Fruit Scheme was introduced to support the distribution of FVs to children together with educational activities to increase consumption and to promote healthy eating habits [36,37]. Within the Italian Accompanying Measures, the Ministry of Agricultural, Food and Forestry Policies funded a teachers’ training program that started in 2014. Consequently, a special Teaching Pack (TP) for teachers was created and tested [38].

The present study aimed to assess the effectiveness of the TP within nutritional interventions led by trained teachers, on 8- to 10-year-old children by evaluating their level of adherence to the MD using the KIDMED test (Mediterranean Diet Quality Index for children and adolescents) a year later.

## 2. Materials and Method

### 2.1. Sampling

**Figure 1:**
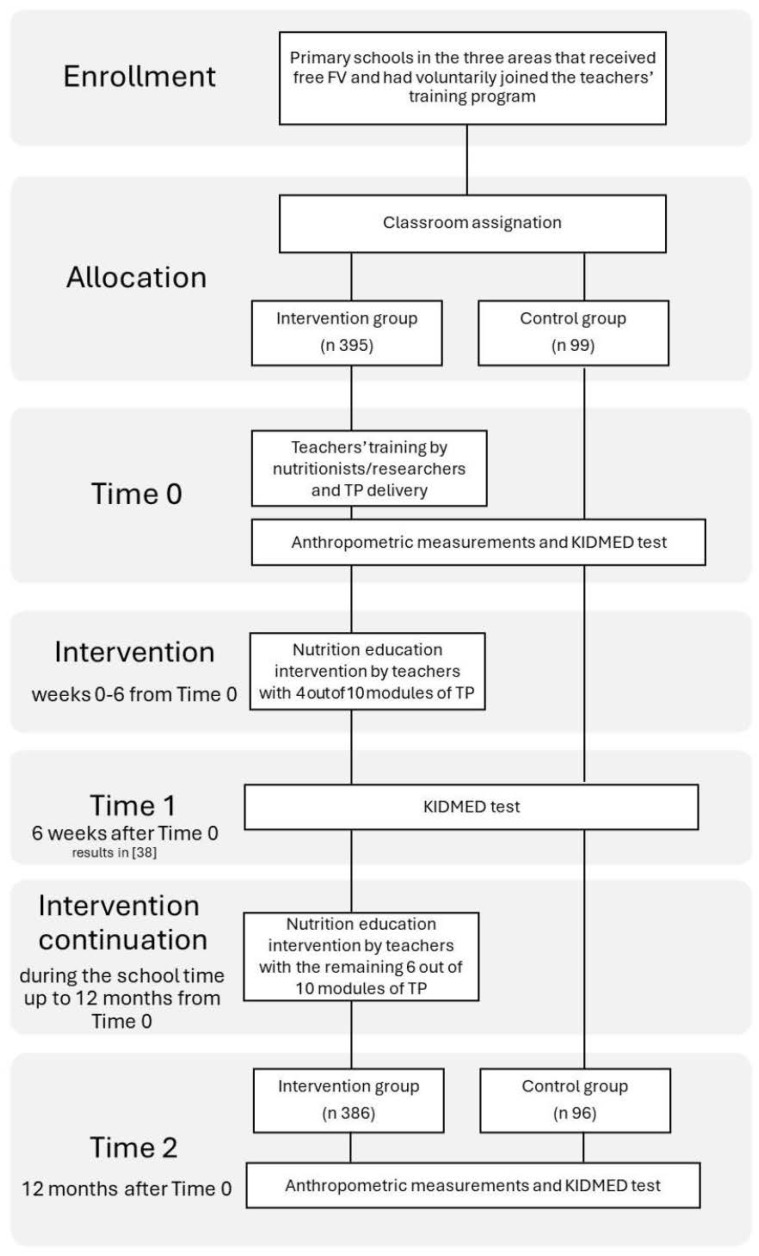
Study flowchart.

The study was carried out in the 2014–2015 and 2015–2016 scholastic years with a pre-post intervention design. Data were collected at baseline, approximately 6 weeks afterwards [38] and after a year (Time 2) as shown in Figure 1. The minimum sample size needed to estimate the percentage of subjects who gave different replies to a key informant question before and after an intervention program was calculated using the result of a past Italian intervention study carried out on a sample of school children (more details in [38]). In the present work, data collected at Time 2 were analyzed in comparison with baseline information. Four hundred and ninety-four children (n = 395-intervention group, n = 99-control group) were included in the analysis. The sample was representative of children in public primary schools taking part in the Italian Fruit School Scheme and getting free FVs. Schools were enrolled based on recruiting fourth class teachers who had voluntarily attended the teachers’ training and agreed to implement the curriculum using the TP according to their timetable. Overall, thirteen schools and 19 teachers participated. Classes where the trained teachers implemented the curriculum (and school children enrolled from these classes) formed the intervention group. The control group subjects were recruited in different fourth classes in the same schools to ensure the homogeneity of the overall sample.

To explore the effectiveness of the TP in various sociocultural contexts, the teachers and therefore the schools were recruited in three Italian geographical areas with a diverse rate of childhood overweight/obesity prevalence, low in Northern Italy, medium in the Center and high in the South, based on the results of the surveillance system “OKkio alla Salute” [39]. In particular, the schools were enrolled in Padua (North), Rome (Center) and Naples (South). All schools and teachers were required not to provide any other educational intervention on nutrition or other health related issues. Also, control group teachers were required not to provide any educational intervention on nutrition or other health related issues.

The study was carried out according to the Declaration of Helsinki. Ethical approval was waived for the study as all the procedures involving the participants were approved by the Boards of the schools involved and were not invasive. Furthermore, parental written consent to participate was required for each child.

### 2.2. Teachers’ Training and Nutrition Intervention

As reported in 2017 [38], at Time 0 nineteen teachers (the intervention group) attended a one-day in-person training carried out by researchers and tailored to give them tools to improve children’s intake of FVs. The main themes were nutritional education and health, the organoleptic quality of FVs, how and when to consume FVs, storage and seasonality. Afterwards, the teachers were given the TP and were required to carry out a self-structured nutritional intervention (choosing at their own discretion when to perform it, based on their time availability and sensitivity) in their classrooms for approximately a month, employing 4 selected modules out of 10 in the TP. Modules include activities to be done both at school and at home and are tailored to improve the intake of FVs and acquire healthy food patterns and lifestyle via ludic activities. Each module consists of a target, an introduction, a launch-story, an activity to be done at school together with the class (for example, to make a scoreboard with all the names of the children, to record any different vegetable and fruit brought to school and eaten in the classroom by each child in a given period of time, usually two weeks, at the end of which a winner is picked based on the number of items eaten), and some homework to involve parents, too, as recommended [40,41,42] (e.g., to write a list of all kinds of fruits and vegetables available at home in a given day, together with parents). They are cross-curricular, involving different subject areas (language, science and so on). The first four modules, regarding the key themes for the children and for the Accompanying Measures, were: “FRUTTOMBOLA”; “DIAMO TEMPO AL TEMPO”; “FRUTTA IN TUTTI I SENSI”; “FRUTTUOSA MERENDA” (about the importance of variety and seasonality, the use of the five senses, and snacks that are not only good for the health but also appetizing). Teachers in the control group were not trained and did not get the TP. Both intervention and control subjects were part of the School Fruit Scheme and got the same FVs.

After the second collection of data, the intervention group teachers were asked to go on with the educational interventions in their classrooms delivering the remaining six modules: “LA MISURA CHE FRUTTA”; “I CINQUE COMANGIAMENTI”; “COLAZIONE MAI PIU’ SENZA”; “FRUTTI DELLA TERRA”; “MUOVITI MUOVITI”; “SIAMO ALLA FRUTTA” (about the importance of right portions, healthy dietary habits, breakfast, pulses, physical activity, water and food waste) following the same procedures.

### 2.3. Anthropometric Measurements

Weight and height measurements were performed on all intervention and control children by two standardized observers, according to WHO guidelines [43] at Time 0 and Time 2. The height measurement was carried out using a SECA 214^TM^ stadiometer (Hamburg, Germany), to the nearest 0.1 cm. Bodyweight was assessed using a SECA 872^TM^ electronic scale (Hamburg, Germany), to the nearest 50 g. To measure body weight, a validated simplified procedure was used [44], the same one applied in primary school children by the Italian surveillance system “OKkio alla SALUTE”. This procedure simplifies measurements and can also help encourage participation by children and parents. This procedure requires that the weight of the children is measured with the clothes on (each marked on the measurement form), instead of wearing only light underwear as required by the recommendations (but always checking that any pockets are empty and still taking off their shoes, any heavy clothing, such as a jacket or sweater and belt); subsequently, in data processing, the estimated weight of each item worn during measurement is subtracted from the measured weight. The anthropometric measurements were carried out in the morning at school with the child fasting (or after only a light breakfast) and maintaining confidentiality with each child. Body mass index (BMI) was obtained from weight (in kg) divided by height squared (in m). Weight status classification was performed through the age- and sex-specific BMI cut-offs of the International Obesity Task Force (IOTF) [45].

### 2.4. Assessment of MD Patterns

The level of adherence to the MD was evaluated by the KIDMED test (Mediterranean Diet Quality Index for children and adolescents). It is composed of 16 “yes” or “no” questions (e.g., “Takes a fruit or fruit juice every day?”; “Has fresh or cooked vegetables regularly once a day?”) [46]. Queries with a negative connotation in regard to the MD are given a value of −1 and the ones with a positive one +1. The overall score ranges from −4 to 12 and there are three levels of adherence: ≥8 high; 4–7, average; ≤3, low [47]. A single nutritionist administered the KIDMED test like a direct interview of all the subjects. As the study was carried out within the “School Fruit Scheme” program, fruit juice was defined as fresh squeezed juice in the interview.

### 2.5. Statistical Analysis

All of the statistical analyses were performed using the computer software IBM SPSS Statistics, version 20.0 (SPSS Inc., Chicago, IL, USA). Statistical tests for normality were conducted for continuous variables, and the data were found to be consistent with a normal distribution. Means, medians and standard deviations (SD) were calculated, and Student’s *t*-test was used to compare means between the groups. Categorical variables were presented as absolute and relative frequencies (percentages). The distributions of sex and BMI categories between the intervention and control groups, and BMI categories by sex and geographical area in the intervention group, were compared using the Pearson chi-squared test for independence. The percentages of subjects with low, average and optimal adherence were calculated in the overall sample, and by sex, main geographical area and BMI class. Differences in overall adherence to the MD before (Time 0) and after (Time 2) the intervention, and by geographical area, sex and ponderal status, were analyzed through contingency tables and Mc Nemar–Bowker test for correlated proportions. To detect differences in the responses to the yes/no questions of the KIDMED test before and after the intervention and by sex and geographical area, the McNemar test (2 × 2 tables) for correlated proportions was used.

All statistical tests were two-tailed, with *p* ≤ 0.05 as the threshold for statistical significance.

## 3. Results

The characteristics of the subjects in both groups at Time 2 are shown in Table 1, while those at baseline are in the Supplemental Table S1. At Time 2 there was a drop-out of nine intervention group children and three control group children, due to their moving to other schools.

22.8% and 8.0% of the subjects in the intervention group were overweight and obese, respectively. The differences between the two sexes (overweight: boys 25.3%, girls 20.5%; obesity: boys 10.2%, girls 6.0%, *p* = 0.121) were not significant. A significant association was found between ponderal status and geographical area (*p* = 0.000) (Table 2), with a higher percentage (19.4%) of obese children in the South (Center 4.3%, North 4.0%).

The control group showed similar characteristics to the intervention group, except for age (*p* = 0.016). No significant differences were observed in the KIDMED score between the intervention and control groups at Time 0 or at Time 2 (Table 1).

The total percentages of optimal and low adherence in the intervention group (Table 3) were 24.4% and 15.0% at Time 0 and raised to 43.3% and 3.9%, respectively, one year after the intervention (*p* < 0.0001).

The percentage of subjects with optimal adherence significantly improved both in females (25.5% vs. 49.5%, *p* < 0.0001) and males (23.1% vs. 36.6%, *p* < 0.0001) in all the areas (North 29.6% vs. 48.0%, *p* < 0.0001; Center 28.8% vs. 43.6%, *p* < 0.0001; South 10.2% vs. 36.7%, *p* < 0.0001), and in all the ponderal classes (Thinness-Normalweight 25.1% vs. 44.9%, *p* < 0.0001; Overweight 25.0% vs. 39.8%, *p* < 0.003; Obesity 16.1% vs. 38.7%, *p* = 0.014) (Table 3).

In regard to the control group, at Time 0 the total percentages of subjects with low, average and optimal adherence were 19.4%, 61.2% and 19.4% and changed to 16.3%, 55.1% and 28.6% at Time 2, (*p* = 0.267) (data shown in Supplementary Table S2).

**Figure 2:**
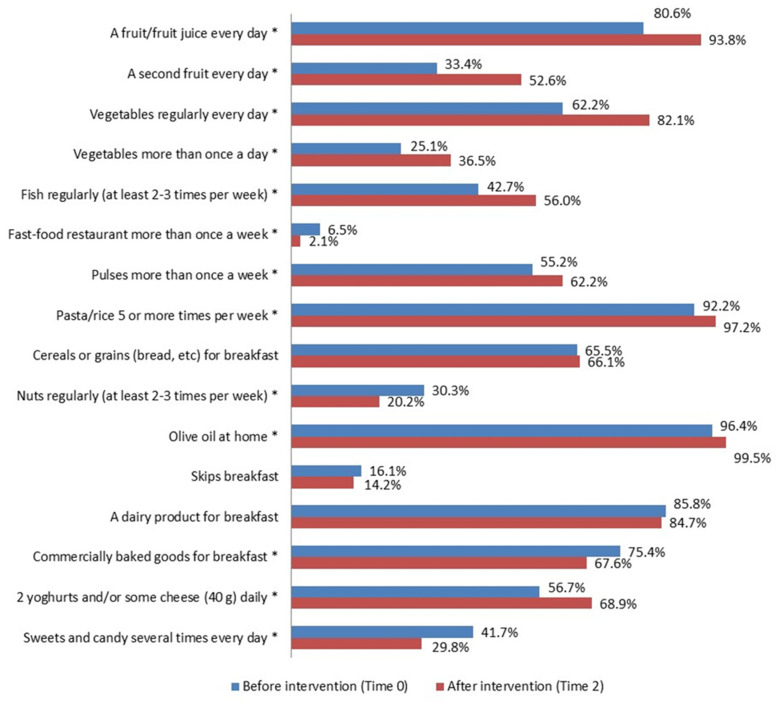
KIDMED test before (Time 0) and after the intervention (Time 2) in the intervention group (%). * *p* ≤ 0.05, McNemar test for differences between correlated proportions, before (Time 0) and after the intervention (Time 2).

**Figure 3:**
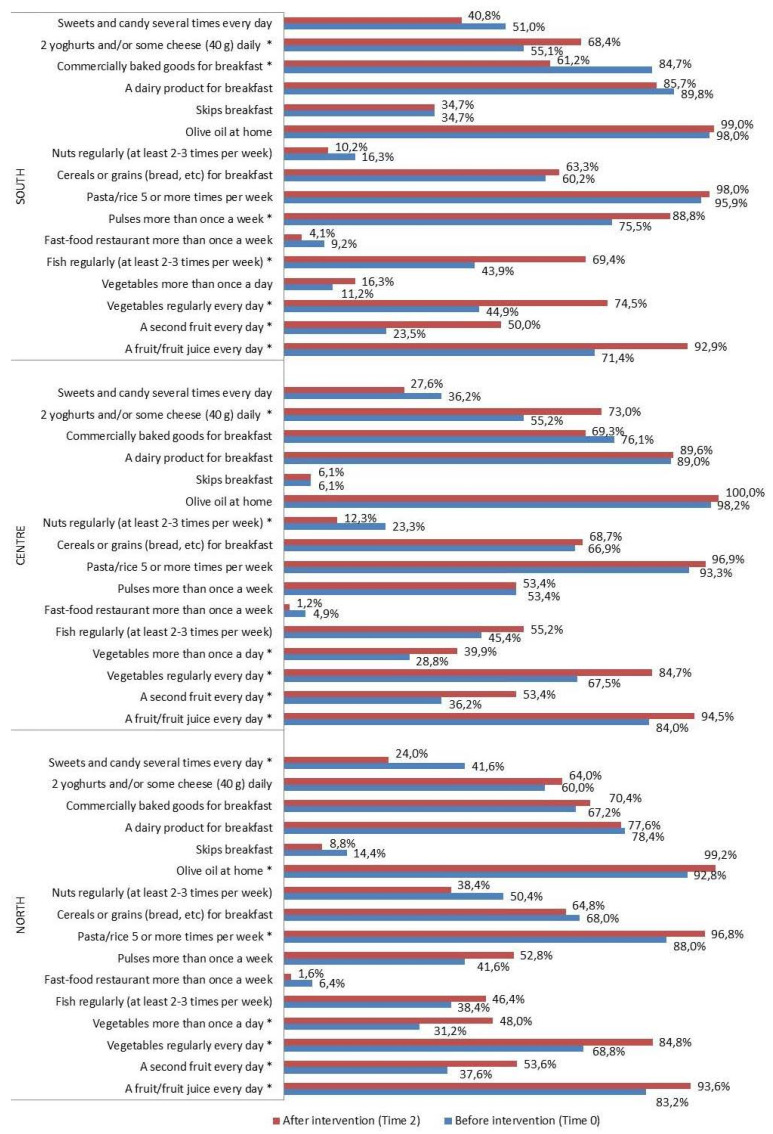
KIDMED test before (Time 0) and after the intervention (Time 2), by geographical area (%) in the intervention group. * *p* ≤ 0.05, McNemar test for differences between correlated proportions, before (Time 0) and after the intervention (Time 2).

**Figure 4:**
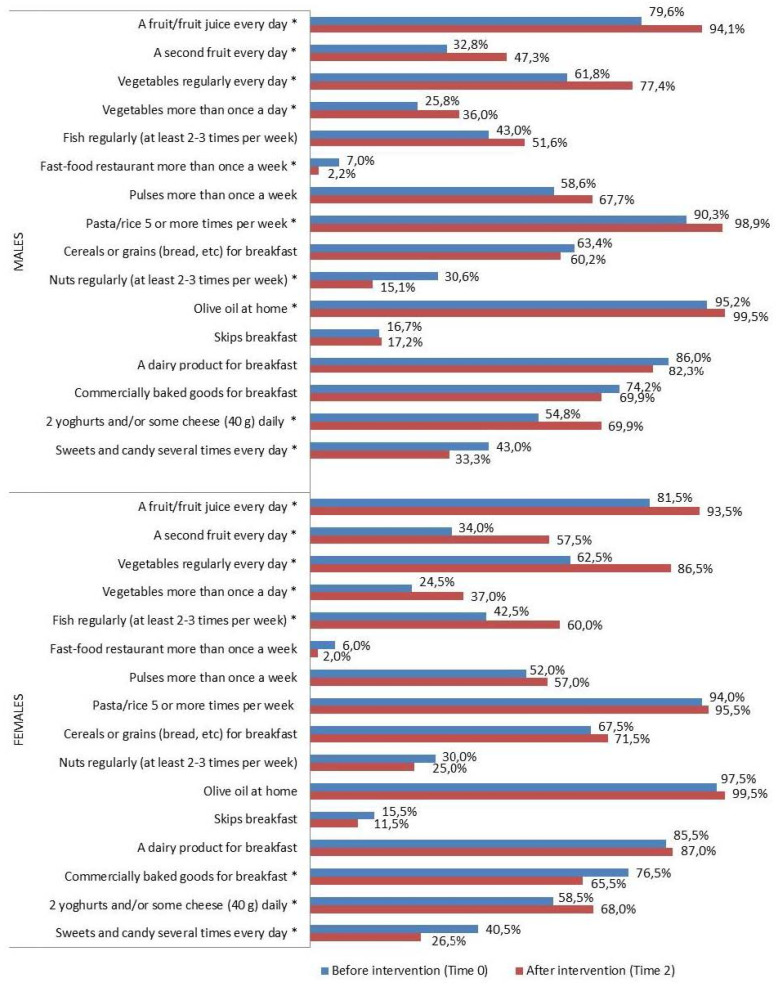
KIDMED test before (Time 0) and after the intervention (Time 2), by sex (%) in the intervention group. * *p* ≤ 0.05, McNemar test for differences between correlated proportions, before (Time 0) and after the intervention (Time 2).

With regard to eating habits (Figure 2, Figure 3 and Figure 4), a significant improvement was detected in the percentage of intervention group subjects who ate (Time 0 vs. Time 2): -*a fruit or fruit juice every day*, in the total intervention group (80.6% vs. 93.8%, *p* < 0.0001), in both sexes and all geographical areas; -*a second fruit every day* in the total intervention group (33.4% vs. 52.6%, *p* < 0.0001), in males and females, and in all geographical areas; -*fresh or cooked vegetables regularly once a day* in the total intervention group (62.2% vs. 82.1%, *p* < 0.0001), in both sexes and all geographical areas; -*fresh or cooked vegetables more than once a day* in the total intervention group (25.1% vs. 36.5%, *p* < 0.0001), in males, females, North and Center; -*fish regularly 2–3 times a week* in the total intervention group (42.7% vs. 56.0%, *p* < 0.0001), in females and in the South; -*pulses more than once a week* in the total intervention group (55.2% vs. 62.2%, *p* = 0.025), in the South; -*pasta/rice 5 or more times a week* in the total intervention group (92.2% vs. 97.2%, *p* = 0.02), in males and in the North; -*olive oil at home* in the total intervention group (96.4% vs. 99.5%, *p* = 0.004), in males and in the North; -*2 yogurts and/or some cheese (40 g) daily* in the total intervention group (56.7% vs. 68.9%, *p* < 0.0001), in males and females, in the Center and South. A significant decrease was also seen in the percentage of children who used -*fast food restaurant more than once a week* in the total intervention group (6.5% vs. 2.1%, *p* = 0.001) and in males; -*commercially baked goods for breakfast* in the total intervention group (75.4% 67.6% *p* = 0.01), in females and in the South; -*sweets and candy several times every day* in the total intervention group (41.7% vs. 29.8% *p* < 0.0001), in males and females, and in the North; -*nuts regularly 2–3 times a week* in the total intervention group (30.3% vs. 20.2% *p* = 0.001), in males and in the Center.

**Figure 5:**
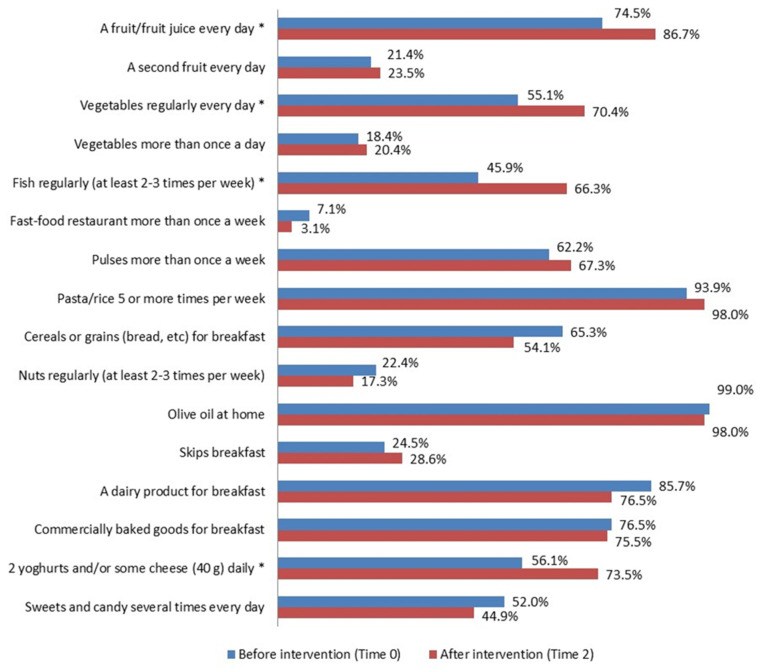
KIDMED test before (Time 0) and after the intervention (Time 2) (%) in the control group. * *p* ≤ 0.05, McNemar test for differences between correlated proportions, before (Time 0) and after the intervention (Time 2).

An improvement in some eating habits (Figure 5) supported by statistical significance was also observed in the control group (Time 0 vs. Time 2). Those of interest for the assessment were: -*a fruit or fruit juice every day* (74.7% vs. 86.7%, *p* = 0.008); -*fresh or cooked vegetables regularly once a day* (55.1% vs. 70.4%, *p* = 0.012); -*fish regularly 2–3 times a week* (45.9% vs. 66.3%, *p* = 0.003); -*2 yogurts and/or some cheese (40 g) daily* (56.1% vs. 73.5%, *p* = 0.005).

## 4. Discussion

The aim of the study was to evaluate the long-term effects of the use of the TP within nutritional interventions carried out by previously trained teachers on children’s adherence to the MD within the Italian Accompanying Measures to the School Fruit Scheme.

The poor rate of subjects with high adherence in the children sampled at Time 0, above all in Southern Italy [31], due to a low-quality diet with a scarce intake of fresh fruit and nuts, vegetables and legumes, key foods of the MD, is in line with the clear tendency to abandon the Mediterranean eating patterns in the pediatric stage, as observed in past studies in Mediterranean areas [24,32,33,34,35]

A significant association between adherence in the pre-intervention and geographical area was observed, in agreement with past research conducted in Southern Italy, describing very small percentages of optimal adherence [48,49]. In fact, in the South the percentage of subjects with optimal adherence was lower and that of children with low adherence higher. A year later, interesting improvements in all the adherence rates were observed in all the geographical areas, as a result of the favorable changes in all the eating patterns of the intervention group subjects. It is noteworthy that schools and teachers were required not to provide any other educational interventions on nutrition or other health related issues during the intervention with the TP. In particular, in the South the percentage of low adherers decreased from 27.6% to 5.1% and that of high adherers improved from 10.2% to 36.7%. In addition, a significant increase of the percentages of the subjects who had a fruit or fruit juice every day; fresh or cooked vegetables regularly once a day; fish regularly 2–3 times a week; pulses more than once a week and 2 yogurts and/or some cheese (40 g) daily was detected.

In the North, almost all the children increased their frequency of intake of fruit or fruit juice every day and olive oil at home. More than half of the subjects had fresh or cooked vegetables regularly once a day. There was also an increase in the consumption frequency of 2 yogurts and/or some cheese (40 g) daily.

As for the Center, more than half of the subjects reported eating a second fruit daily, and a significant improvement was also observed in the percentage of those eating vegetables more than once a day.

With regard to sex, a significant reduction in the rate of subjects with low adherence and concurrently an increase of the percentages of high adherers were found in both females and males. It is noteworthy that the improvement of the eating patterns in females was detected not only in the higher frequency of daily intake of FVs (in both classes “a second fruit every day” and “vegetables more than once a day”), as analogously detected in males, but it was also accompanied by a parallel significant reduction in the intake frequency of sweets, candy and commercially baked goods for breakfast (Figure 4), suggesting, as in past studies, a further improvement in the eating habits, possibly due to substituting sweet snacks/foods with FV-based alternatives, as recommended in the modules [50,51].

The same could also apply to the significant reduction in the pattern of consuming nuts regularly (at least 2–3 times a week) found in the whole intervention group and in the South at Time 2. It is important to notice that the decrease in the frequency of nuts consumption is not in accordance with the best Mediterranean food patterns.

FVs and all the other key foods of the MD provide many health benefits, but unfortunately, as already mentioned, there is a trend toward the abandonment of the MD by younger people, so these findings are very positive.

Finally, regarding ponderal status, the highest percentages of overweight and obesity were observed in the South of Italy, as expected. The results are in line with those of the surveillance system OKkio alla SALUTE [52] and of other Southern European Mediterranean areas, like Greece and Spain [53]. It is noteworthy that a year after the intervention there was a very positive significant improvement of the percentages of adherence in all the ponderal classes. In particular, the percentages of low and high adherers changed as follows at Time 0 vs. Time 2: overweight class (low 15.9% vs. 4.5%) (high 25.0% vs. 39.8%); obesity class (low 32.3% vs. 3.2%) (high 16.1% vs. 38.7%). However, given the small sample sizes in the Central Italy and Obesity subgroups, some results should be considered with caution.

Given the importance of FV within a healthy and high-quality diet, many interventions carried out in schools are intended to improve the consumption of these foods, using different educational components [54,55,56,57]. Some entail tasting experiences [58] or games that teach healthy food patterns by playing [59]; school gardening activities [60] or in recent years, digital approaches, in particular, the Internet, telehealth, mobile apps and so on [61]. All of these approaches seem to be effective and have favorable impacts on children, but being mainly performed by expert people they are usually deliverable only at regional levels [62,63].

Among the main strengths of the study, which is being carried out within a national project aimed at involving about one million children annually, is the training of teachers all around the country to provide them with tools, pedagogical instruments and materials, to deliver the nutritional program when and as they like without external interventions, tailoring the intervention as best fits with the school context, and considering the children’s educational context and environment, which can differ a lot between regions [64,65,66]. Besides, training teachers nationally allows us to reach all schools despite their territorial characteristics.

The methodology developed for this intervention study is also a strength. In fact, it was chosen to prevent overloading the everyday schedule of already innumerable school activities with too many extra tasks and to promote implementation, overcoming the barrier of lack of and/or insufficient teachers’ time and taking into consideration their central role as key agents for the promotion of health and nutrition within schools [67,68,69,70]. Before starting to deliver the nutrition education program to children, the intervention group teachers were trained by researchers in the promotion of FV consumption and other healthy dietary habits using evidence-based content and were given the appropriate information about the modules. As previously observed, it is fundamental that teachers get sustained or trained to feel confident and competent when delivering nutritional content, and the training could significantly increase teachers’ self-efficacy in doing it [70,71]. In fact, preservice teachers are usually provided with scarce training in nutrition issues during their studies [72] and may not have the competence, reason or ability to deliver evidence-based nutritional education [73]. As in past research [71,74], the cross-curricular approaches, incorporating nutrition education into everyday teaching practices, integrating it into other subjects, and pleasant and ludic activities embedded in the intervention led by trained teachers showed very encouraging and successful results. As for the intervention length, though even short-term interventions seemed to achieve healthy changes [38,75,76], long interventions, as in the present study, were found to be required to obtain improvements in healthy food habits and behavioral changes. A minimum of 6 months is suggested [71,77,78].

Other strengths of the study were measuring body weight and height of the subjects and not using self-reported data [79] and administering the KIDMED test in a face-to-face interview conducted by a nutritionist, as this could help prevent inadequate replies due to scarce/lack of attention or memory failure when self-completing a questionnaire [80]. This method allowed the interviewer to refer directly to the fruit juice (named as an item on the KIDMED test) as fresh squeezed juice; both the TP and the teachers’ training sessions promote the consumption of fresh fruit (mainly whole fruit).

Finally, as reported in other studies [74,75], it is fundamental to include control groups. In this study, the smaller number of control children, compared to the number of intervention children, could be a limit, but it was necessary to ensure as much homogeneity of characteristics as possible between the two groups, so all the children were enrolled in the same schools. For ethical reasons, in schools involved in a European Program like the School Fruit Scheme, it was not possible to raise the number of control subjects by moving subjects from the intervention group whose teachers agreed to attend the training course. The statistical comparison between intervention and control in subgroups was not allowed due the number of control children. Similar rates of subjects in the intervention and control groups were taken into consideration in previous research within the European School Fruit Scheme [81].

The positive achievements observed could also, at least in part, be the result of the compliance tendency of subjects toward the interviewer, and this could be another limit. In any case, the results show that the children have learned the importance of FV and the other Mediterranean foods/principles.

Schools are the perfect setting to promote healthy eating habits, and this study, despite its limitations, provides useful findings about the Italian School Fruit Scheme. Particularly, it sustains past evidence showing that multicomponent interventions combining availability of FVs with a nutritional program embedded in the school curriculum and led by trained teachers, improve the intake of key healthy foods [55,68,71,82]. As for the control group, significant changes in some of the dietary habits were observed, suggesting, like in previous studies, that just the free FV supply alone in schools can have a favorable effect [67,83,84,85,86]. However, these changes can be enhanced when well-structured educational interventions support the availability of a free FV supply.

Finally, although not supported by formally gathered data, the teachers involved in the intervention spontaneously reported that the children were very enthusiastic and involved in the activities proposed and showed more eagerness to taste and consume FVs, both at school and at home.

## 5. Conclusions

The findings from this study add to past studies suggesting that schools can be the ideal setting for promoting healthy food patterns in children by accompanying the free distribution of FVs with a nutrition education intervention tailored to teachers’ and children’s needs, easy to implement and sustainable over the long term. A program led by trained teachers, incorporated into the school’s curriculum and activities with cross-curricular approaches that is also pleasant for children (being based on ludic activities) offers good chances of success. The positive improvements observed in the MD adherence rates in all the geographical areas and classes of ponderal status were particularly noteworthy in the South and among overweight/obese children, where the improvements are most needed. This represents an interesting achievement and support for researchers and future interventions, policymakers and pedagogues as to educational strategies and public health investment.

As also indicated by past studies, nutritional education should be a regular and usual component of school activities, not competing with core subjects, for the betterment of children’s short- and long-term health.

## Figures and Tables

**Table 1:** Sample characteristics and KIDMED scores in intervention and control groups at TIME 2.

	Intervention (n = 386)			Control (n = 98)				
	Mean	Median	SD	Mean	Median	SD		*p*-Value
Age (years)	10.7	10.7	0.3	10.6	10.7	0.4		0.016 ^a^*
Weight (kg)	41.3	39.8	10.1	41.0	38.9	10.1		0.761 ^a^
Height (cm)	145.7	145.6	7.2	144.4	143.4	7.3		0.114 ^a^
BMI (kg/m^2^)	19.3	18.9	3.6	19.5	18.7	3.7		0.639 ^a^
KIDMED score at baseline	5.9	6.0	2.2	5.4	5.5	2.2		0.061 ^a^
KIDMED score after the intervention	6.4	7.0	2.2	6.0	6.0	2.4		0.176 ^a^
**BMI Category ^§^**	**n**	**%**		**n**	**%**		**Chi-Square**	***p*** **-Value**
Thinness/Normalweight	267	69.2		63	64.3		0.955	0.620 ^b^
Overweight	88	22.8		25	25.5			
Obesity	31	8.0		10	10.2			
**Sex ratio** (% females)	200	51.8		52	53.1		0.049	0.825 ^b^

BMI: body mass index; SD standard deviation. ^a^ Student’s *t*-test; ^b^ Pearson chi-square test; * Effect size of difference between the two means resulted to be small; **^§^** by [45].

**Table 2:** Intervention group characteristics by sex and geographical area at TIME 2.

	Boys (n = 186)			Girls (n = 200)				North (n = 125)			Center (n = 163)			South (n = 98)			
	Mean	Median	SD	Mean	Median	SD	*p*-Value	Mean	Median	SD	Mean	Median	SD	Mean	Median	SD	*p*-Value
Age (years)	10.7	10.7	0.3	10.8	10.7	0.4	0.389 ^a^	10.8	10.8	0.3	10.8	10.8	0.4	10.7	10.7	0.3	*0.014*
Weight (kg)	42.0	40.0	10.7	40.6	39.5	9.5	0.180 ^a^	41.5	41.0	10.2	39.8	38.9	9.4	43.6	40.8	10.7	*0.015*
Height (cm)	145.0	144.9	6.7	146.4	146.8	7.5	0.063 ^a^	146.3	146.7	7.5	145.9	146.1	7.0	144.6	144.8	7.0	*0.193*
BMI (kg/m^2^)	19.8	19.3	3.9	18.8	18.3	3.3	0.006 ^a^	19.2	18.7	3.5	18.6	18.1	3.3	20.6	19.8	3.9	*0.000*
**BMI Category ^§^**	**n**	**%**		**n**	**%**	**Chi-Square**	***p*** **-Value**	**n**	**%**		**n**	**%**		**n**	**%**	**Chi-Square**	***p*** **-Value**
Thinness/Normalweight	120	64.5		147	73.5	4.218	0.121 ^b^	90	72.0		123	75.5		54	55.1	25.843	0.000
Overweight	47	25.3		41	20.5			30	24.0		33	20.2		25	25.5		
Obese	19	10.2		12	6.0			5	4.0		7	4.3		19	19.4		

BMI: body mass index; SD standard deviation. ^a^ Student’s *t*-test; ^b^ Pearson chi-square test; ^§^ by [45] Cole & Lobstein 2012.

**Table 3:** Overall MD adherence before (Time 0) and after the intervention (Time 2), in the intervention group, by sex, geographical area and ponderal status.

	Before Intervention (Time 0)						After Intervention (Time 2)						
	Low		Average		High		Low		Average		High		
**Geographical area**	n	%	n	%	n	%	n	%	n	%	n	%	*p* Value *
North	16	12.8%	72	57.6%	37	29.6	7	6.6	58	46.4	60	48.0	0.002
Center	15	9.2	101	62.0	47	28.8	3	1.8	89	54.6	71	43.6	<0.0001
South	27	27.6	61	62.2	10	10.2	5	5.1	57	58.2	36	36.7	<0.0001
**Sex**													
Male	30	16.1	113	60.8	43	23.1	9	4.8	109	58.6	68	36.6	<0.0001
Female	28	14.0	121	60.5	51	25.5	6	3.0	95	47.5	99	49.5	<0.0001
**Ponderal status**													
Thinness/Normal weight	34	12.7	166	62.2	67	25.1	10	3.7	137	51.3	120	44.9	<0.0001
Overweight	14	15.9	52	59.1	22	25.0	4	4.5	49	55.7	35	39.8	0.003
Obesity	10	32.3	16	51.6	5	16.1	1	3.2	18	58.1	12	38.7	0.014
**Total intervention group**	58	15.0	234	60.6	94	24.4	15	3.9	204	52.8	167	43.3	<0.0001

* *p*-value from McNemar–Bowker test for differences in correlated proportions, before (Time 0) and after the intervention (Time 2).

## Data Availability

The raw data supporting the conclusions of this article will be made available by the authors on request.

## Supplementary Materials

The following supporting information can be downloaded at: https://www.mdpi.com/article/10.3390/nu16132057/s1, Table S1: Sample characteristics in intervention and control groups (standard deviation SD) before intervention (Time 0); Table S2: Adherence before (Time 0) and after the intervention (Time 2), in the control group, by sex, geographical area, ponderal status.
