# Supplementary material for: Improvement of Adherence to the Mediterranean Diet through a Nutrition Education Teaching Pack for Teachers within the “School Fruit Scheme” Program: An Italian Long-Term Trial in School Children
Source: Nutrients. 2024 Jun 27;16(13):2057. doi: 10.3390/nu16132057 (PMC11243712; doi:10.3390/nu16132057)
Supplement: Supplementary file 1 [file nutrients-16-02057-s001.zip › nutrients-3012356-supplementary.pdf]

Table S1. Sample characteristics in intervention and control groups at TIME 0.

	Intervention (n=386)			Control (n=98)				
	**mean**	**median**	**SD**	**mean**	**median**	**SD**		**p-Value**
**Age (years)**	9.6	9.6	0.3	9.6	9.6	0.4		0.331[a]
**Weight (kg)**	36.0	34.4	9.0	35.7	33.7	8.3		0.751[a]
**Height (cm)**	138.8	138.6	6.5	138.1	137.8	6.8		0.367[a]
**BMI (kg/m2)**	18.5	18.0	3.6	18.6	17.8	3.2		0.883[a]
**BMI category§**	**n**	**%**		**n**	**%**		**chi-square**	**p-Value**
**Thinness/Normalweight**	264	68.4		66	67.3		0.944	0.624[b]
**Overweight**	85	22.0		25	25.5			
**Obese**	37	9.6		7	7.1			

BMI: body mass index; SD standard deviation
a Student's t-test
b Pearson chi-square test
§ by Cole & Lobstein 2012

Table S2. Adherence before and after the intervention at Time 2, in the control group, by sex, geographical area, ponderal status

	Before intervention						After intervention						
	Low		Average		High		Low		Average		High		
	n	%	n	%	n	%	n	%	n	%	n	%	p-Value*
**Geographical area**													
**North**	6	23.1	17	65.4	3	11.5	4	15.4	18	69.2	4	15.4	n.d.
**Centre**	3	12.0	15	60.0	7	28.0	4	16.0	11	44.0	10	40.0	n.d.
**South**	10	21.3	28	59.6	9	19.1	8	17.0	25	53.2	14	29.8	n.d.
**Sex**													
**Male**	11	23.9	25	54.3	10	21.7	7	15.2	27	58.7	12	26.1	0.409
**Female**	8	15.4	35	67.3	9	17.3	9	17.3	27	51.9	16	30.8	0.144
**Ponderal status**													
**Thinnes/Normalweight**	11	17.5	39	61.9	13	20.6	11	17.5	34	54.0	18	28.6	0.515
**Overweight**	5	20.0	15	60.0	5	20.0	1	4.0	16	64.0	8	32.0	0.082
**Obesity**	3	30.0	6	60.0	1	10.0	4	40.0	4	40.0	2	20.0	0.513
**Total**	19	19.4	60	61.2	19	19.4	16	16.3	54	55.1	28	28.6	0.267

* p-Value from McNemar-Bowker test for differences in correlated proportions, before and after the intervention
